# Examining the Building Blocks of Health Behavior Change in Rheumatology Rehabilitation: A Theory-Driven Qualitative Study

**DOI:** 10.2147/JMDH.S472713

**Published:** 2024-07-29

**Authors:** Gunnhild Berdal, Ingvild Kjeken, Anita Dyb Linge, Ann Margret Aasvold, Kjetil Tennebø, Siv Grødal Eppeland, Anne Sirnes Hagland, Guro Ohldieck-Fredheim, Helene Lindtvedt Valaas, Ingvild Bø, Åse Klokkeide, Maryam Azimi, Turid N Dager, Anne-Lene Sand-Svartrud

**Affiliations:** 1 Health Services Research and Innovation Unit, Center for Treatment of Rheumatic and Musculoskeletal Diseases (REMEDY), Diakonhjemmet Hospital, Oslo, Norway; 2 Muritunet Rehabilitation Centre, Valldal, Ålesund, Norway; 3 Meråker Rehabilitation Centre, Meråker, Norway; 4 Valnesfjord Health Sports Centre, Valnesfjord, Norway; 5 Department of Physiotherapy, Sørlandet Hospital, Arendal, Norway; 6 Haugesund Hospital for Rheumatic Diseases, Haugesund, Norway; 7 Vikersund Rehabilitation Centre, Vikersund, Norway; 8 Department of Rehabilitation, Hospital for Rheumatic Diseases, Lillehammer, Norway; 9 Rehabilitering Vest Rehabilitation Centre, Haugesund, Norway; 10 REMEDY Patient Advisory Board, Diakonhjemmet Hospital, Oslo, Norway

**Keywords:** rheumatic diseases, musculoskeletal diseases, rehabilitation, behavior therapy, qualitative research, patient engagement

## Abstract

**Purpose:**

To identify and describe behavior change techniques (BCTs) used in rehabilitation for patients with rheumatic and musculoskeletal diseases (RMDs), according to their own perceptions. Further, to examine patients’ descriptions of their capability, opportunity, motivation, and readiness for health behavior change.

**Patients and Methods:**

Patients were adults in need of specialized, multidisciplinary rehabilitation services due to inflammatory rheumatic disease, systemic connective tissue disease, or fibromyalgia / chronic widespread pain. Semi-structured interviews of 21 patients were analyzed with deductive qualitative content analysis applying three theoretical frameworks: the Behavior Change Technique Taxonomy, the transtheoretical model and stages of change, and the capability, opportunity, and motivation model of behavior.

**Results:**

Forty-six BCTs aggregated within 14 BCT groups were identified used by either patients, healthcare professionals (HPs), or both. Goals and planning, feedback and monitoring, social support, shaping knowledge, repetition and substitution were most frequently used to facilitate behavior change. Twenty patients had reached the action stage and made specific lifestyle changes concerning more than half of their goals. Concerning other goals, 6 of these patients reported to be contemplating behavior change and 15 to be preparing for it. The rehabilitation process appeared to strengthen capability, opportunity, motivation, and the desired behaviors. Patient-reported barriers to behavior change were connected with restrictions in physical capability resulting from an unpredictable and fluctuating disease course, weakened motivation, and contextual factors, such as lack of access to healthcare support and training facilities, and high domestic care burden.

**Conclusion:**

The rehabilitation process seemed to strengthen individual and contextual prerequisites for behavior change and facilitate the use of required techniques and engagement in the desired behaviors. However, patients with RMDs may need prolonged support from HPs to integrate healthy lifestyle changes into everyday life. The findings can be used to optimize rehabilitation interventions and patients’ persistent engagement in healthy behaviors.

## Introduction

Rheumatic and musculoskeletal diseases (RMDs) are leading causes of disability,^1^ with affected patients representing the largest group in need of rehabilitation worldwide.^2^ Multidisciplinary rehabilitation for RMDs is a highly complex intervention that consists of various health-promoting components and behavior change techniques (BCTs) aiming to help patients adhere to healthy behaviors and cope with the consequences of chronic disease.^3^ Evidence exists that such rehabilitation leads to better health, function, and quality of life,^4–8^ but the effects are modest and diminish over time.^9^,^10^

The UK Medical Research Council guidance^11^ recommends broadening the focus of complex intervention research beyond the binary question of effectiveness, emphasizing the need for greater attention to the conditions needed to produce behavior change, considering the underpinning theory and the context of implementation. Improved understanding of what works and how, and under what circumstances, can facilitate optimization of rehabilitation interventions and ultimately improve patient health.^11^

Recent advances in behavioral science have provided tools to identify and describe the content of complex behavior change interventions, including BCTs, conceptualized as their presumed active components.^12–14^ The BCT Taxonomy version 1 consists of a number of distinct, consensually agreed BCTs which provide a basis for specifying intervention content using a consistent and scientifically shared terminology. The transtheoretical model of health behavior change^15^ adds a temporal dimension, in line with the common experience that most healthy behaviors require time to become habitual. The model posits that behavior change moves through a series of stages, which, if identified, allow supportive interventions to be tailored to individual needs.^15^ A third theoretical framework: the capability, opportunity, motivation - behavior model^16^,^17^ brings together both individual and contextual factors necessary for behavior change to occur, applying a broader outlook on what might influence behavior change outcomes. However, work remains to explore whether prerequisites for behavior change described in such frameworks are present and used in clinical practice and patients’ individual behavioral processes.

Rehabilitation should be evaluated by those who receive it to optimize service delivery.^18^,^19^ However, there is a paucity of research exploring patients’ perspectives as a driver for quality improvement in RMD rehabilitation.^19^ This has implications for how current rehabilitation meets the needs of this patient group. Patients’ views on what influences health behavior change in a rehabilitation process can provide important information that can be used to improve both rehabilitation interventions and the outcomes of such rehabilitation. Therefore, the aim of this study was to identify and describe BCTs used in rehabilitation for patients with RMDs, according to their own perceptions. Furthermore, the study aimed to examine patients’ descriptions of their capability, opportunity, motivation, and readiness for health behavior change during the rehabilitation process.

## Methods

### Study Design

The study was designed as a qualitative descriptive study where data from individual semi-structured patient interviews were analyzed using qualitative content analysis with a deductive approach.^20–22^

### Study Participants

This study was nested within a larger study, in which eligible patients had to be aged ≥ 18 years and have undergone 2–4 weeks of inpatient multidisciplinary rehabilitation due to inflammatory rheumatic disease, systemic connective tissue disease, osteoarthritis, fibromyalgia/chronic widespread pain, or nonspecific low back, neck, or shoulder pain (persistent for > 3 months). In the current study, we included patients from the largest diagnostic groups, which were inflammatory rheumatic disease, systemic connective tissue disease, and fibromyalgia/chronic widespread pain. Exclusion criteria were cognitive impairment, severe psychiatric disorder(s), and inability to understand and speak Norwegian, as the larger study involved use of Norwegian versions of patient-reported outcome measures collected several times during the first year after admission to specialized rehabilitation.

### Setting, Sampling and Recruitment

The study was conducted within the context of a stepped-wedge cluster randomized trial [The BRIDGE-trial: ClinicalTrials.gov NCT03102814] implemented at eight rehabilitation centers in secondary healthcare, located across all health regions of Norway. Patients were included to the main study in the period from August 2017 to August 2018 and followed for 1 year. The participants in the current study were recruited from the intervention group of the BRIDGE-trial^23^ by local study coordinators at each center in the period from February 2018 to June 2018. Purposive sampling was employed^24^ to ensure inclusion of patients with varied sociodemographic backgrounds at various time points in their rehabilitation follow-up course, who were assumed to contribute useful information and insights to illuminate the study aims. Twenty-two patients were invited to participate, 21 of whom accepted and were included. All participants received a multidisciplinary rehabilitation program^23^ consisting of five evidence-based components underpinned by health behavior change theory: structured goal setting,^25^,^26^ action and coping planning,^26^ digital self-monitoring of progress,^27^ tailored follow-up support,^28^,^29^ and use of motivational interviewing^30^,^31^ in goal setting and follow-up calls. Goal setting and action planning were carried out collaboratively by the individual patients and their rehabilitation team upon admission to rehabilitation and before discharge to home. The patients monitored their own progress by reporting goal status and health outcomes at five time points (admission, discharge, and 2, 6, and 12 months after admission), which generated a digital graph that provided visual feedback. All patients received one mandatory telephone follow-up call delivered 1 month after discharge by their primary contact at the rehabilitation center, and were offered an additional 3 calls (optional) within 6 months after discharge to support goal-directed efforts and health behavior change. The program was added to the traditional rehabilitation programs delivered at the eight rehabilitation centers and tailored to the individual patients’ needs. Details on the intervention content and delivery mode have been published elsewhere.^23^

### Data Collection

Sociodemographic data (age, gender, primary diagnosis, disease duration, comorbidities, educational level, employment, and civil status) were collected on admission to rehabilitation by means of a digital questionnaire.

Qualitative data were collected in semi-structured interviews at a site chosen by the patient, 3–9 months after rehabilitation discharge. The interviews took place in person, were audio-taped, and carried out by one of two experienced interviewers (ALSS, ASH) who applied the same interview guide. The questions addressed the patients’ experiences with receiving the rehabilitation program during the stay and the continued rehabilitation process after discharge. Average interview duration was 60 minutes (range 35–75 minutes). The audio recordings were transcribed verbatim and imported into the QSR NVivo for Windows software (Release 1.6.1).

### Theoretical Frameworks

Successful rehabilitation requires actual behavior change to take place. Behaviors can be those of healthcare professionals (who implement evidence-based practice), or those of patients (who pursue rehabilitation goals or adhere to health advice),^32^ or both. To investigate whether important factors for behavior change were present in the rehabilitation process of our patient informants, we adopted three theoretical frameworks.

### The Behavior Change Technique Taxonomy (version 1) (BCTTv1)

The BCTTv1^14^ outlines 93 techniques clustered into 16 groups that can be used alone or in combinations to facilitate behavior change. BCTs are defined as the smallest observable and replicable components of behavior change interventions, representing the active ingredients expected to bring about behavior change.^14^,^33^ Goal setting, feedback on behavior, and social support are examples of BCTs. The BCT taxonomy is accepted internationally by multiple disciplines and provides a framework for specifying and evaluating behavior change interventions that can be used across different behaviors and contexts.^14^,^34^

### The TransTheoretical Model and Stages of Change (TMM)

The TMM suggests that behavior change unfolds over time through a series of stages.^15^,^35^ The stages are precontemplation, contemplation, preparation, action, maintenance, and termination. To progress from one stage to another, specific behavior change strategies need to be applied at each stage. In precontemplation and contemplation people are typically resistant or ambivalent and not ready to take immediate action. However, they may be receptive to consciousness-raising and motivational influence. In preparation and action people are ready for action-oriented behavior change interventions, and tend to turn more to commitments, conditioning, environmental controls, and social support to progress towards maintenance or termination.^15^ The TTM provides a means for tailoring health behavior change interventions to individual patients’ levels of knowledge, motivation, and readiness for change, while also accounting for relapse prevention.^36^

### The Capability, Opportunity, and Motivation Model of Behavior (COM-B)

The COM-B model posits that three conditions must be in place for someone to engage in a particular behavior: capability, opportunity, and motivation.^16^,^17^ Capability is the individual’s physical and psychological capacity to engage in the activity. It includes having the skills, knowledge, strength, and stamina, as well as the capacity for comprehension and reasoning. Opportunity refers to the external factors that facilitate or allow enactment of the behavior. These include physical opportunities (afforded by the environment, such as time, resources, access) and social opportunities (afforded by the cultural environment that influence our thinking). Motivation is the reflective (conscious intentions, plans, choice, evaluations) and automatic (habitual and emotional responses) processes that stimulate and direct behavior. To engage in a behavior, the motivation must be stronger than for any competing behaviors.^16^,^17^ The model incorporates context, which is recognized as crucial to effective implementation of health behavior change interventions.^16^,^37^ The COM-B is a generic model for capturing all factors known to influence behavior change and is widely recognized to inform intervention design, explain findings, and guide qualitative data analysis.^38^

#### Data Analysis

The transcripts were analyzed using a theory-driven deductive qualitative content analysis.^20^,^22^ Three coding frameworks were established, based on the BCTTv1,^14^,^39^ the TMM,^15^ and the COM-B model of behavior,^16^,^17^ respectively. All pertaining categories were defined according to published descriptions,^14–16^,^39^ and entered into the NVivo software. Following a completed open online BCTTv1 training course (www.bct-taxonomy.com) and a pilot testing of the frameworks, the transcribed material was thoroughly reviewed and coded if the content corresponded to the pre-established categories. Coding frequencies were registered and patient quotes collected to anchor the empirical data to the theoretically derived categories. The coding was carried out for each framework separately by the first author (GB) and then repeated to enhance rigor. The coding process was discussed with two co-authors (ALSS, IK) to ensure transparency and trustworthiness.

Coding to the BCT taxonomy was done at the techniques level, then aggregated to the sixteen overarching BCT groups. A distinction was made between BCTs that patients reported using themselves and BCTs used by the HPs who were involved in their rehabilitation process, as perceived by the patients. Illustrative content was entered into tables, together with figures on the incidence of use of BCTs in the sample. Coding to stages of change was linked to text passages about the patients’ rehabilitation goals. Each goal in terms of desired behavior was tabulated and counted, and meaningful content was condensed and linked to one of the six stages corresponding to the patient’s expressed position in the behavioral change process. Coding to the COM-B model had a broader scope, focusing on patient-reported capability, motivation, and opportunity to engage in goal-directed health behavior change. The interviews were carried out in Norwegian. After the analysis, illustrative patient quotes were first translated by the first author (GB), before the quality of the translation was ensured by an English-language editing service.

#### The Research Team

The research team consisted of healthcare professionals of various professional backgrounds (physiotherapists, occupational therapists, clinical psychologist) specialized in rehabilitation, patient research partners, and researchers with longstanding experience in rheumatology rehabilitation research. The research team was supported by a steering group with expertise in physical medicine, general practice, primary and secondary healthcare, and international RMD rehabilitation perspectives.

#### Ethics

The study was approved by the Norwegian Regional Committee for Medical Research Ethics (REK South-East, 2017/665), and conducted in agreement with the Helsinki Declaration and the ICMJ Recommendations for the Protection of Research Participants. Written, informed consent was obtained from all study participants before inclusion in the larger BRIDGE trial. For the current study, the participants gave an additional consent to publication of anonymized responses.

## Results

Background characteristics of the 21 included patients are presented in Table 1. They ranged in age from 32 to 77 years. The majority were female, with longstanding inflammatory rheumatic disease, and several comorbidities. Approximately half had a higher education and/or were employed.

**Table 1:** Sociodemographic Characteristics of the Included Patients (n = 21)

Age, median (range)	56 (32–77)
Female sex, n (%)	17 (81)
Primary diagnosis, n (%)	
Inflammatory rheumatic disease^a^	15 (71)
Chronic widespread pain / fibromyalgia syndrome	4 (19)
Connective tissue disease	2 (10)
Disease duration, years, median (range)	11 (2–39)
Comorbidities, n, median (range)	3 (0–6)
Educational level > 12 years, n (%)	10 (48)
Paid employment, yes, n (%)	11 (52)
Civil status, living with partner, n (%)	13 (62)
Time elapsed since rehabilitation stay, patients, n (%)	
3–4 months	3 (14)
5–6 months	16 (76)
8–9 months	2 (10)

**Note**: ^a^Rheumatoid arthritis, spondyloarthritis, psoriatic arthritis.

### Behavior Change Techniques

**Figure 1:**
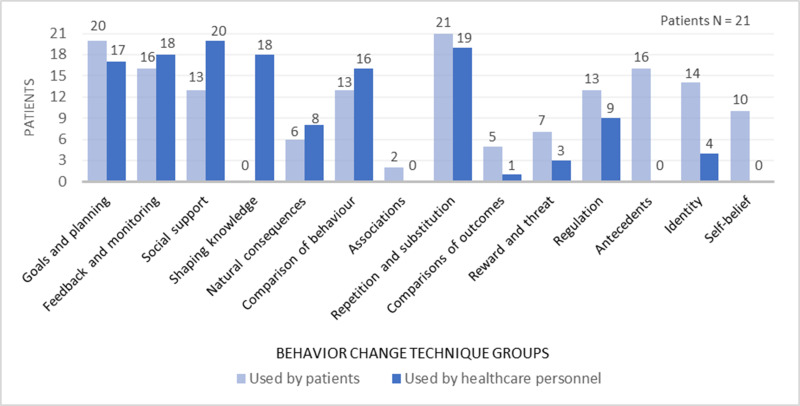
Behavior change techniques (BCT) used by patients while undergoing rehabilitation and their perception of BCTs used by the healthcare personnel involved.

Figure 1 shows the patient-reported BCTs used either by the patients or the HPs with whom they interacted during the rehabilitation process, categorized according to the 16 overarching BCT groups of the taxonomy (BCTTv1).^14^ The BCT groups that were most frequently used by the patients were goals and planning (n=20), feedback and monitoring (n=16), repetition and substitution (n=21), antecedents (n=16), and identity (n=14). The BCT groups most frequently used by the HPs to facilitate the patients’ processes were goals and planning (n=17), feedback and monitoring (n=18), social support (n=20), shaping knowledge (n=18), and repetition and substitution (n=19). Two BCT groups (scheduled consequences and covert learning) were not reported as used by any of the patient informants and therefore omitted from display in Figure 1.

Appendix 1 shows the specific BCTs reported as used, with frequencies of occurrence in the data material and examples of how they were applied during rehabilitation. A total of 46 BCTs were identified. All but one patient (20/21) reported having set written rehabilitation *goals*. The goals covered several health behavior change domains and were largely reviewed and adjusted in collaboration with HPs over the course of rehabilitation (17/20). Most patients (18/21) received *feedback* from their HPs on behavior change efforts, both face-to-face during the rehabilitation stay and by telephone follow-up after discharge. The majority of patients used *self-monitoring* strategies on behavior or outcome of behavior (16/21), such as training diaries, pedometers, timing of activities, and/or a digital graph showing progress on outcomes. *Social support* (general, practical, emotional) was received from HPs (20/21), family, friends, fellow patients, and others (13/21). *Instructions on how to perform* desired behaviors were provided by the HPs to shape new knowledge, either individually or in groups/educational classes (18/21). Repetition was also a dominant approach, as all patients *practiced* and *repetitively rehearsed* the desired behaviors. Moreover, most patients (18/21) received *supervision* from the HPs involved during the rehearsals, including individual tailoring on how to perform the behaviors.

The patients also reported using a series of other BCTs, such as *rewards* (material, social, self-reward) (7/21), *regulation* (emotional, cognitive, pharmacological) (13/21), *antecedents* (restructuring the physical or social environment, adding objects to make desired behavior feasible, and distraction or avoidance of exposure to cues for undesired behavior) (16/21), *identity* (linking new behavior to valued self-identity, self-affirmation, and identification of self as role model, and reframing new activities to personal values) (14/21), *self-belief* (positive self-talk, focus on past success to facilitate implementation of new desired activities) (10/21), and *social comparison* (comparison of own performance to fellow patients’ used as inspiration and motivation for further behavioral change efforts) (13/21) (Appendix 1).

### Stages of Change

**Figure 2:**
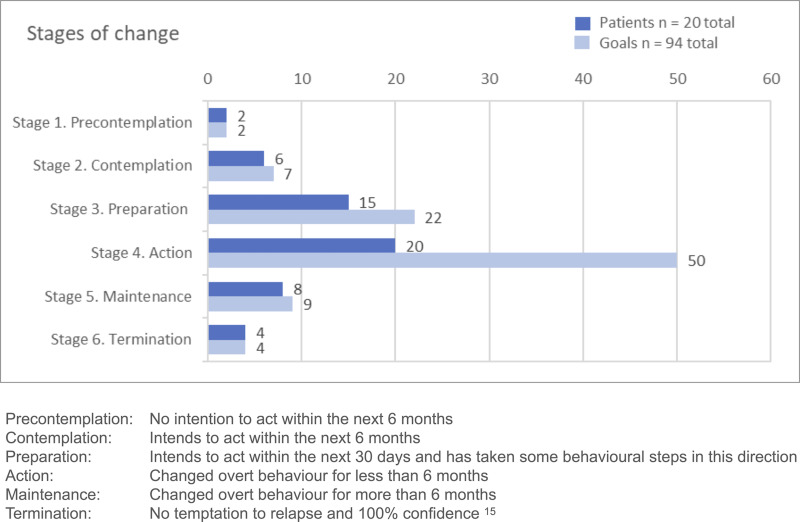
Patient-reported stages of change according to stated health behavior goals.

Figure 2 shows the patient-reported stages of change according to stated rehabilitation goals. One patient had no personal rehabilitation goal(s). The twenty others had between 3 and 6 goals, each covering various domains, such as physical exercise, restitution, healthy diet, weight reduction, smoking cessation, and improvement of sleep, social relations, and work-life participation. All reported having made specific, overt modifications in their lifestyle (stage 4, action) with regard to at least one of their behavior goals. At group level, the patients reported being actively engaged in changing behavior related to more than half of their goals (50/94 goals). However, six patients reported still contemplating some of their goals (stage 2, contemplation), and 15 patients reported preparing to take action regarding several other health behavior goals (stage 3, preparation). Eight patients reported confidently working to prevent relapse of health behavior change (stage 5, maintenance), while four patients reported having reached stage 6 (termination), meaning that a specific task was completed (eg a mountain hiking trip), or that the behavior had become automatic, without experienced temptations (eg successful change of diet). Table 2 shows raw data examples of text coded to the six stages of change.

**Table 2:** The Stages of Change According to the Transtheoretical model^15^ with Examples of Coded Raw Data

Stages	Patient Quotes	Informant No.
Stage 1. Precontemplation	“…she [therapist] thinks that maybe I should have signed up for some training, but that’s not for me. I can’t bear to pay for something that I can’t follow up on, no”.	1
Stage 2. Contemplation	“…and then I’ve been thinking about quitting smoking, but I haven’t gotten there yet”.	10
Stage 3. Preparation	“I know myself too well, that ‘doorstep mile’, it can be horrible, so I know I have to make appointments with someone who says that I have to”.	9
Stage 4. Action	“…now I go every Wednesday to exercise with the group (...), and on Tuesdays and Thursdays it’s me and our eldest daughter; at 6 o’ clock in the morning we go training (...) and then at 7 o’ clock we go swimming (...). Occasionally, we go for a walk on Sunday mornings, and occasionally on other days too, so... so it’s [exercise] at least, four days a week”.	4
Stage 5. Maintenance	“I guess I had a relapse. I ate half a rice chocolate, but then, (...) my body felt so bad (...), huge stomachache. So no, I do not really fancy chocolate anymore (...). I have been without sugar for a couple of months now. (...) For the time being, knock on wood, it’s going very, very, very well, and I can do it”.	17
Stage 6. Termination	“One of my goals was to go up and down [a ski jump hill] which has 1078 steps. I did it twice”.	11

### Capability, Opportunity, and Motivation for Health Behavior Change

**Figure 3:**
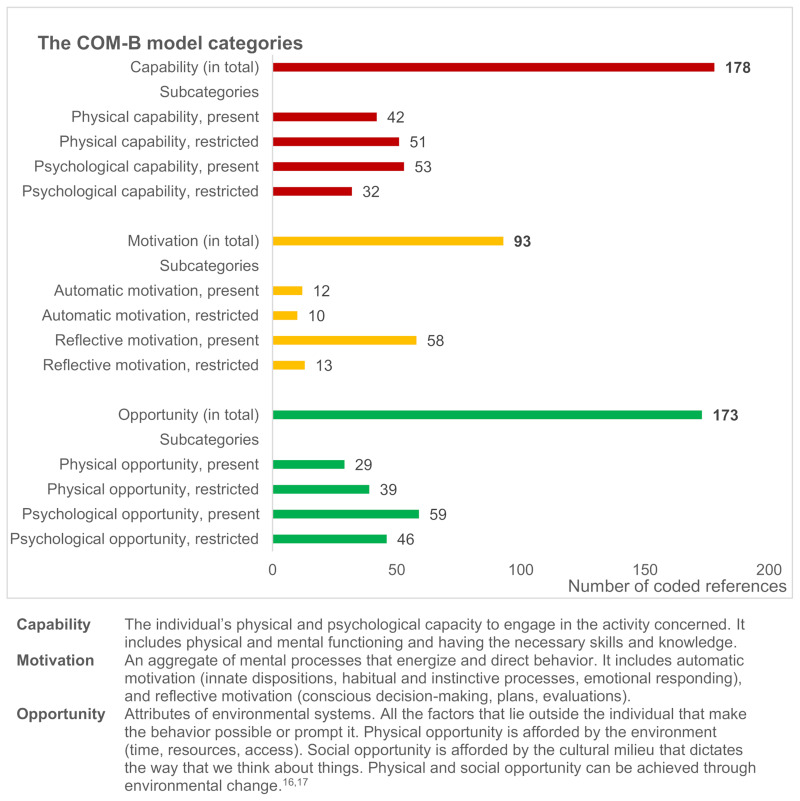
Coding frequencies of patient-reported capability, motivation and opportunity to engage in goal-directed health behavior change during rehabilitation for RMDs, based on the categories provided by the COM-B model, identified across 21 patient interviews.

The patients’ capability, motivation, and opportunity to engage in goal-directed health behavior change were mapped according to the COM-B model (Figure 3). The coding frequencies in Figure 3 represent how often the different components of the model were mentioned in the data material. In the following, we present our interpretations of the meaningful content of the coded material, supported by illustrative patient quotes.

Physical capabilities were often related to exercise practices and improvement in physical fitness, as demonstrated by the following patient quote:

I started to exercise quite a lot because I had a goal to improve my health by training (...). The more I move around, the more I notice: My fitness is better (...); I can walk and talk all the way up the hill. Earlier I had to stop and pause. (Informant 21)

Furthermore, statements indicating the presence of physical capability were frequently accompanied by statements about restrictions in physical capability, which were expressed in terms of adaptation:

I have a lot of trouble walking because I have a very painful sensation under my feet (...) I can’t walk more than a couple of hundred meters... and it’s a bit painful even at rest, but it gets worse when I walk. So that’s why... biking is kind of what works best, physically. (Informant 14)

Similar descriptions of capability were found regarding the ability to work, which was linked to increased self-care and ability to regulate activity level, acquired as a result of the rehabilitation process:

I had one particular goal, which was keeping myself at work (...), and I haven’t been on a sick leave or anything after that, so... I’ve become better at taking care of myself, and at delegating things. I sort of say [to colleagues]; Can you take this? (...) I’ve actually become much better at that, and all the guys at work understand this. (Informant 5)

Reflective motivation (Figure 3), particularly conscious, goal-oriented intentions, seemed to have been strengthened as result of the rehabilitation process. The patients’ motivation was largely described in terms of reflective reasoning related to goals and plans:

…when you set goals, you (...) become more aware of what you really want, what kind of everyday life you truly desire. (Informant 15)

For some, visualizing attaining the goal was helpful to resist temptations:

Every time I felt like having a cigarette, I just pictured [name of mountain peak], and then I thought: No, I’m going there first. Before any cigarette, I’m going to the [mountain]. (Informant 11)

Restrictions in reflective motivation were largely related to ambivalence or resistance towards health behavior change. Some patients had managed to establish new healthy habits, drawing on automatic motivation in their continued efforts. Others experienced a decrease in positive emotional response while trying to continue the activities initiated at the rehabilitation center:

I tried the exercises we did [at the rehabilitation center].., but it didn’t go well. I couldn’t do it. And it just became unpleasant. (Informant 2)

The presence of both motivation and psychological capability were perceived as resources for moving forward with goal-directed activities, yet not always enough to overcome fluctuating restrictions in physical capability:

I’m not uncertain about whether I should do this, or should I cycle, or should I do yoga, or... I’m very clear about what I’m going to do, [I have] very clear goals that aren’t [too] big. It’s just that sometimes, I’m in such [bad] shape that I’m not able to do it. (Informant 14)

Other patients described restrictions in physical and psychological capability as intertwined, impacting on their general ability to cope:

I feel that I have much poorer mental capacity, and psychologically, right, when you’re constantly tired and drained, experiencing pain and poor sleep, and feel that you fall short, you know, everything becomes cumbersome. (Informant 3)

In particular, pain appeared to impact on both physical and psychological capability:

For me, it goes very hand in hand, the mental aspect and the pain. I am very strong when I am pain-free, and then I am very weak when I am in pain. Then I’m like, there are tears, and I take everything very heavily. (Informant 16)

Amidst the complexity of having a fluctuating and unpredictable condition, receiving attention and advice from HPs throughout the rehabilitation process was experienced as reinforcing the motivation for and effects of personal efforts:

A focus on progression, I haven’t had that before. I noticed that I found it meaningful, and that it made me experience greater joy in it [a personal training program]. (Informant 3)

The importance of seeing results from one’s own work, and having a form of control, was emphasized as highly valuable:

There was one thing... such an important realization (...) and a huge extra bonus was that all that growth, everything I achieved, was thanks to myself! There was no treatment I had received, no injection, no tablets, no physical therapy, nothing else that had contributed to that improvement in function. (ibid)

Regarding physical opportunities (Figure 3) several external factors were reported to facilitate health behavior change, such as work life adjustments, access to training facilities, self-monitoring devices, medical adjustments, use of technical aids, and nice weather. Conversely, restricted physical opportunities were characterized by insufficient healthcare support and training facilities within the local community, long travel distances, lack of appropriate technical aids, and harsh weather conditions. As examples of social opportunities, the patients highlighted valuable support from family, friends, and other social groups, goodwill from managers at work, and understanding attitudes from colleagues. Restricted social opportunities included complex social obligations in close relationships that competed with opportunities for self-care, major caregiver tasks that competed with personal rehabilitation activities, summer holidays that interrupted routines, having a cigarette-smoking spouse while trying to quit, and a workplace culture with frequent access to cookies while trying to lose weight.

For several patients, the rehabilitation process seemed to have positively impacted on health behavior change by providing initial security, mastery experiences, and motivation to continue being active in a purposeful way. Conceptualized within the COM-B model, the rehabilitation process provided opportunity (physically and socially), motivation (through eg goal setting and emotional responding to feedback), and capability (through increasing knowledge, self-efficacy, skills, and physical fitness):

…during those three weeks [at the rehabilitation center], you know, it felt like I went from 0 to 100 in terms of fitness. It’s been a long, long time since I felt this security and yes, that I became positive about physical activity again without being afraid, you know. You realize that yes, I can do this without anything [adverse] happening. That’s really good. (Informant 9)

## Discussion

In this study, we applied three key theoretical frameworks for understanding and supporting behavior change to explore the use of BCTs in rehabilitation for RMDs according to patients undergoing rehabilitation, as well as their individual and contextual prerequisites to engage in health behavior change.

Forty-six BCTs aggregated within 14 BCT groups were identified across 21 patient interviews. The BCT groups most frequently used by patients were goals and planning, feedback and monitoring, repetition and substitution, antecedents, and identity. The BCT groups perceived to be most frequently used by HPs, overlapped with those used by the patients in terms of goals and planning, feedback and monitoring, and repetition and substitution, but also included social support and shaping knowledge. Our findings indicate that goal setting and action and follow-up planning were largely collaboratively developed between the patients and the HPs involved, as part of the rehabilitation process. Furthermore, we found that thorough practice and repetition of individually valued rehabilitative activities were accompanied by professional guidance, self- and external monitoring, and prolonged supportive feedback from HPs, relatives, colleagues, fellow patients, diaries, wearables, and other tools. The findings are consistent with previous research on the use of BCTs in health behavior change interventions for RMDs, which reports a large number of BCTs applied in rehabilitation, with the most common techniques being goal setting, problem solving, self-monitoring, feedback, and social support.^40–43^

In addition, the patients reported using a variety of other BCTs to progress in their rehabilitation process. These included self-regulating, rewarding, and assertive techniques, inspirational social comparison, and restructuring of surroundings to facilitate desired behavior. Previous research has shown that using many BCTs may increase the effectiveness of health behavior change interventions,^40^,^44^ but the optimal content and mode of delivery are still unknown.^23^,^41^,^43^ Two BCT groups (scheduled consequences and covert learning) were not reported used by the patients. These groups include BCTs focused on arrangements for (factual and imaginary) punishment and reward,^39^ which were not part of the intervention package received by the patients.^23^ In cases where the patients nevertheless reported use of rewards as motivation for behavioral change, this was coded under the BCT group rewards and threats. This, in addition to patient preferences, may explain why our results show no use of the two mentioned BCT groups in the present study.

Based on our patient interviews, it appears that empowering patients to become flexible and adaptable agents in their own rehabilitation process could be a core component of behavior change interventions. The ability to adjust coping responses to changing circumstances has been found to reduce the negative impact of pain and disability on the psychological wellbeing of patients with RMDs who experience fluctuating and unpredictable disease courses.^45^,^46^ Accordingly, HPs should support and encourage the continuation of such practices.

The patient-reported stages of change showed that all patients with expressed rehabilitation goals had reached the action phase (stage 4) and made specific lifestyle changes related to more than half of their goals. Furthermore, forty percent had reached the maintenance phase (stage 5) for at least one goal, indicating that the majority of patients were well underway with lifestyle changes. The results further revealed that the patients had several goals covering different life domains. Changing multiple behaviors simultaneously represents a particular challenge, placing demands on both patients and HPs. According to the TMM, action is the most challenging stage, and taking action on two or more behaviors at the same time can be overwhelming.^15^ This may explain why a considerable proportion of patients reported that they were still contemplating and/or preparing for behavior change regarding some goals. The TMM suggests that people in the contemplation and preparation stages are receptive to using various cognitive, affective, and evaluative processes to move forward to action, which can be done by adopting either sequential (changing one behavior at a time) or integrative approaches.^15^ From an HP perspective, acknowledging that patients have multiple goals necessitating different behavior changes may suggest that distinct types of guidance are required for each goal. This may also indicate that some goals should be prioritized while others may be temporarily deferred to avoid conflicting processes for the individual.

Conceptualized within the COM-B model, the rehabilitation process seemed to bolster capability, opportunity, and motivation. Capability improved through exercise, skills training, education, guidance, improved self-care, and mastery experiences. Physical opportunities were provided through the rehabilitation facilities while social opportunities were provided by support from HPs and fellow patients and from workplace adjustments and support from significant others after discharge. Motivation was strengthened through goal setting and feedback, including the feedback derived from experiencing the results of one’s own efforts. The dynamics of the COM-B model^16^,^17^ were illustrated by individual patient stories of how attentive support and advice (O) throughout the rehabilitation process boosted motivation (M) for continued exercise efforts (B), resulting in fitness progression (C), which further strengthened motivation (M) and capability (C) to perform the desired behavior (B). In other words, the prerequisites of healthy behaviors (capability, opportunity, motivation) changed over time as a result of a complex interplay between the components.^17^ In some phases, the rehabilitation process was mainly driven by the available opportunities. In other phases, the driving factors were primarily related to the patients’ motivation, pursuit of goals, and progression in capability through behavioral practice. Consequently, from a HP’s perspective, it may not be enough to provide unilateral guidance towards only one dimension of the COM-B model. Good rehabilitation practice should contribute parallel attention to patients’ capacity, motivation, and contextual possibilities, and meet these with appropriate therapeutic strategies, to increase the probability that the desired behavior change is actually implemented and maintained.

The patients often presented their capabilities in a dual manner, incorporating both their capacities and limitations, thereby demonstrating their adaption to and management of the constraints resulting from their condition. Still, a main finding was that several patients intermittently experienced their physical capability as so restricted that it hindered their participation in the desired activities, despite their having the opportunity, motivation and psychological capacity. This underlines the importance of close medical monitoring to ensure satisfactory disease control. Furthermore, offering extended and intensified support from HPs throughout the rehabilitation trajectory may enable patients to overcome such fluctuating capability restrictions. However, recent studies have highlighted the scarcity of follow-up care following specialized RMD rehabilitation, particularly as concerns complex multidisciplinary health services.^47^,^48^ According to theories of behavior change maintenance,^29^ limited resources may become depleted in the presence of stressors, such as flare-ups with increased disease activity and high symptom burden, affecting capability. When resources are low, self-regulation may fail and result in relapse and setbacks. However, people tend to maintain behaviors if they possess sufficient psychological and physical resources and are in a supportive environment,^29^ all of which should be facilitated in a rehabilitation process.

This study is strengthened by the thorough research methodology, in which three widely recognized theoretical frameworks were applied systematically to examine fundamental components of health behavior change in rehabilitation. Study limitations may include the complexity of the frameworks and data material, which were managed by one single coder. However, to maximize dependability and required coder skills, extensive training and piloting were conducted prior to the study, with subsequent recoding performed as part of the primary analysis, yielding results consistent with the initial findings. The deductive strategy suggests the possibility of overlooking relevant content that did not align with the predefined categories. Moreover, the rehabilitation context of a Scandinavian healthcare system may influence the transferability of the findings. However, the comprehensive and transparent reporting should provide readers with sufficient information to assess the applicability of the results in other contexts.

## Conclusion

We conclude that patients with RMDs, supported by HPs, use a broad spectrum of BCTs while undergoing rehabilitation. Goals and planning, feedback and monitoring, and repetition and substitution were most frequently applied to facilitate health behavior change. Based on established health behavior change theory and associated tools, HPs can promote a diverse range of techniques for patient self-support throughout the rehabilitation process. This may facilitate a more precise and personalized use of BCTs in rheumatological rehabilitation, thereby potentially improving health outcomes. Furthermore, our findings emphasize the importance of paying attention to external factors, such as physical facilities and supportive environments to facilitate the implementation and maintenance of desired behaviors. In cases where health behavior change is desired within several life domains, HPs should tailor their strategies to patients’ readiness for change for each specific goal. Our findings suggest that the rehabilitation process served to strengthen the foundation for behavior change in terms of patients’ capabilities, opportunities, and motivation, while also fostering flexible, adaptable, and persistent engagement in the desired healthy behaviors. However, patients with RMDs may need continuous support from HPs for a longer period of time to optimize and integrate healthy lifestyle changes into their everyday life.

## Data Availability

No further data will be shared.
